# Chitosan-Modified AgNPs Efficiently Inhibit Swine Coronavirus-Induced Host Cell Infections via Targeting the Spike Protein

**DOI:** 10.3390/biom14091152

**Published:** 2024-09-13

**Authors:** Dongliang Wang, Caiyun Yin, Yihan Bai, Mingxia Zhou, Naidong Wang, Chunyi Tong, Yi Yang, Bin Liu

**Affiliations:** 1 College of Biology, Hunan University, Changsha 410082, China; dongliangwang@hnu.edu.cn (D.W.); yincaiyun@hnu.edu.cn (C.Y.); sw_tcy@hnu.edu.cn (C.T.); 2 Hunan Provincial Key Laboratory of Protein Engineering in Animal Vaccines, College of Veterinary Medicine, Hunan Agricultural University, Changsha 410128, China; byh248950700@stu.hunau.edu.cn (Y.B.); mingxiazhou@stu.hunau.edu.cn (M.Z.); naidongwang@hunau.edu.cn (N.W.)

**Keywords:** silver nanoparticles, coronavirus, chitosan, disulfide bonds, ROS/p53 signaling pathway

## Abstract

The COVID-19 pandemic, caused by severe acute respiratory syndrome coronavirus 2 (SARS-CoV-2), has filled a gap in our knowledge regarding the prevention of CoVs. Swine coronavirus (CoV) is a significant pathogen that causes huge economic losses to the global swine industry. Until now, anti-CoV prevention and control have been challenging due to the rapidly generated variants. Silver nanoparticles (AgNPs) with excellent antimicrobial activity have attracted great interest for biosafety prevention and control applications. In this study, we synthesized chitosan-modified AgNPs (Chi-AgNPs) with good biocompatibility to investigate their antiviral effects on swine CoVs. In vitro assays showed that Chi-AgNPs could significantly impaired viral entry. The direct interaction between Chi-AgNPs and CoVs can destroy the viral surface spike (S) protein secondary structure associated with viral membrane fusion, which is caused by the cleavage of disulfide bonds in the S protein. Moreover, the mechanism showed that Chi-AgNPs reduced the virus-induced apoptosis of Vero cells via the ROS/p53 signaling activation pathway. Our data suggest that Chi-AgNPs can serve as a preventive strategy for CoVs infection and provide a molecular basis for the viricidal effect of Chi-AgNPs on CoVs.

## 1. Introduction

Infectious diseases have led to a worldwide health crisis and have had a huge impact on the public health system and global environmental and economic development globally [1]. Approximately 20% of global mortality is caused by infectious diseases [1,2], of which 80% is attributed to viral infections [3]. Particularly, the acute respiratory diseases of the coronavirus disease 2019 (COVID-19) caused by the serve acute respiratory syndrome coronavirus 2 (SARS-CoV-2) outbreak in 2019 resulted in a global health crisis [4,5]. Despite possessing several antiviral drugs and vaccines, the continuous generation of variants and side effects of long-term use also pose challenges to traditional antiviral therapies [6].

Coronaviruses (CoVs) belong to the family of *Coronaviridae* and are widely found in humans and animals. To date, based on the phylogenetic relationships and genomic criteria, CoVs can be divided into four genera: *Alphacoronavirus* (α-CoV), *Betacoronavirus* (β-CoV), *Gammacoronavirus* (γ-CoV) and *Deltacoronavirus* (δ-CoV) [7]. Currently, six CoVs have been identified in pigs: porcine epidemic diarrhea virus (PEDV), transmissible gastroenteritis virus (TGEV), porcine respiratory CoV (PRCV), and swine acute diarrhea syndrome CoV (SADS-CoV) belonging to α-CoV, porcine hemagglutinating encephalomyelitis virus (PHEV) belonging to β-CoV and porcine deltacoronavirus (PDCoV) belonging to δ-CoV [7,8]. Among them, PEDV and TGEV are considered the major CoVs that cause very high mortality in piglets and are responsible for huge economic losses in the swine industry, which has been circulating in pig populations for several decades [9]. Conventional bivalent vaccines based on whole virus antigens have been widely used for the prevention of PEDV and TGEV infections [10]; however, the continuous variants have brought huge challenges to immune efficacy and remain an extensive prevalence of virus infection in pig populations [11,12]. Thus, there is an urgent need to develop effective antiviral agents to control the CoV infections in pigs.

**Scheme 1:**
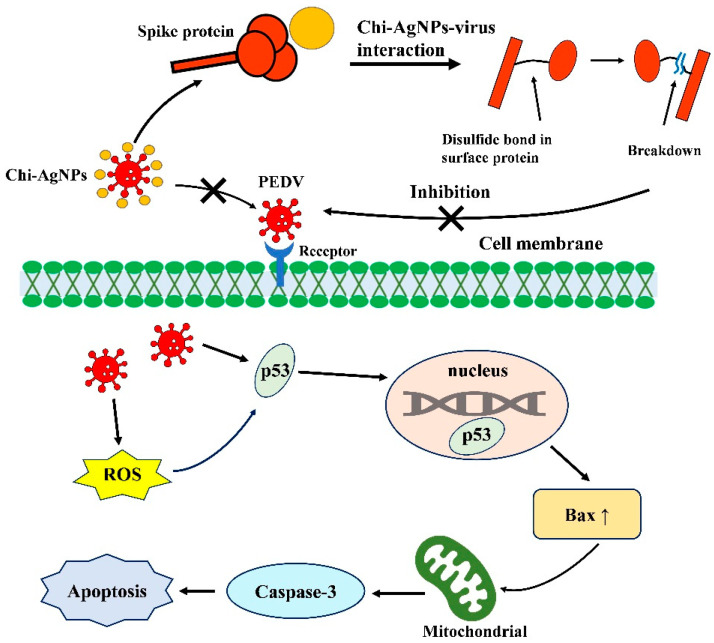
Schematic illustration of PEDV treated with Chi-AgNPs. Chi-AgNPs interact with PEDV surface protein (Spike protein) and cleaves their disulfide bonds. Chi-AgNPs-treated PEDV exhibit lower infectivity to cells. Inhibitory effects of Chi-AgNPs on PEDV-induced host cell infection and ROS/p53 signaling activation. The proposed model shows that PEDV causes cell apoptosis through activation of ROS and p53-mediated apoptotic pathway, which could be inhibited by Chi-AgNPs.

Advances in nanotechnology have played a vital role in the prevention, treatment, and diagnosis of viruses through the design of nanoparticles [13,14]. Metal nanomaterials exhibit great potential for combating viruses because of their attractive physicochemical and biological properties as well as their unique structure and size [15]. Among these, silver nanoparticles (AgNPs) with antibacterial, antifungal, and antiviral activities have been extensively investigated. In particular, AgNPs can be used as antiviral reagents against influenza virus [16,17], respiratory syncytial virus (RSV) [18], human immunodeficiency virus (HIV) [19], hepatitis B virus (HBV) [20], and herpes simplex virus (HSV) [21]. Chitosan is a type of alkaline polysaccharide characterized by non-toxicity, good biocompatibility, and biodegradability. Chitosan-modified AgNPs (Chi-AgNPs) have great potential in the biomedical applications owing to their excellent antiviral effects. However, the antiviral properties of Chi-AgNPs against CoVs are limited. This study aimed to investigate the inhibitory effects of Chi-AgNPs on CoVs and their potential molecular mechanism between Chi-AgNPs and viruses. In this study, PEDV was used as a model for CoVs to evaluate the antiviral effects of the Chi-AgNPs (Scheme 1). The inhibitory effects of Chi-AgNPs on PEDV-induced cell infection via ROS/p53 signaling activation were determined. More importantly, the molecular mechanism underlying the viricidal effects of Chi-AgNPs and viruses was elucidated.

## 2. Materials and Methods

### 2.1. Materials

AgNO_3_, NaBH_4_, NaOH, chitosan, and citrate were purchased from Sigma Aldrich (St. Louis, MO, USA); trypsin, Dulbecco’s modified Eagle’s medium (DMEM), fetal bovine serum (FBS), penicillin–streptomycin, 4,6-diamidino-2-phenylindole (DAPI), FITC-labeled donkey anti-mouse IgG, and HRP-labeled goat anti-mouse IgG were purchased from Invitrogen (Invitrogen, Carlsbad, CA, USA); Cell Counting Kit-8 (CCK-8), RIPA lysis buffer and BCA protein assay kit were purchased from Beijing Solarbio Science & Technology Co., Ltd. (Solarbio, Beijing, China); a reactive oxygen species (ROS) kit, mitochondrial membrane potential assay kit with JC-1 and Tris-(2-carboxyethyl) phosphine hydrochloride (TCEP) were purchased from Beyotime Biotechnology (Beyotime, Shanghai, China); an annexin V-FITC/PI apoptosis detection kit was obtained from Shanghai Yeasen biotech Co., Ltd. (Yeasen, Shanghai, China); GAPDH (0.6 mg/mL), β-actin (1 mg/mL), p53 (0.6 mg/mL), Bax (0.8 mg/mL), and caspase 3 (0.6 mg/mL) polyclonal antibodies were obtained from Proteintech (Proteintech, Chicago, IL, USA); anti-PEDV spike (S) and nucleocapsid (N) protein mouse polyclonal antibodies (1 mg/mL) were kept in our lab.

### 2.2. Preparation and Characterization of Chi-AgNPs

For the synthesis of chitosan-modified AgNPs (Chi-AgNPs), an AgNO_3_ solution (10 mM, 10 mL) was mixed with a chitosan solution (5 mg/mL, 4 mL) dissolved in 1% acetic acid, which was followed by stirring for 10 min at room temperature. Then, cold NaBH_4_ (0.1 M, 1 mL) was added to the mixture solutions, the color changed from white to yellow brown, and then the resultant solution was subjected to adjusting the pH value of 6.0 by adding 420 µL of NaOH (1 M), which was followed by adding water to a final volume of 21.6 mL. For comparison, citrate-modified AgNPs (AgNPs) were prepared as follows: briefly, 4 mL of AgNO_3_ solution (50 mM) was mixed with 0.2 mL of citrate solution (1 M), which was followed by addition of water to a volume of 99 mL and stirring for 30 min. Finally, 1 mL of cold NaBH_4_ (0.1 M) was slightly added to the resultant solution, which was stirred for 30 min in the dark.

The morphology of the AgNPs was observed using high-resolution transmission electron microscopy (TEM, JEOL JEM-2100, Tokyo, Japan). The size distribution and zeta potential of the AgNPs were characterized using a Zetasizer (Nano ZS90 instrument, Malvern, UK). The ultraviolet-visible (UV-vis) absorbance of AgNPs was measured using a DU800 spectrometer (Beckman Coulter Inc., Brea, CA, USA). Fourier transform infrared (FT-IR) spectra were measured using a Thermo-Nicolet Nexus 6700 FT-IR spectrometer (TENSOR27, Thermo Fisher Scientific Inc., Waltham, MA, USA). Elemental analysis was performed using an X-ray photoelectron spectrometer (XPS, Thermo Fisher Scientific K-Alpha, Greater Mumbai, MA, USA).

### 2.3. Cell Culture and Viruses

African green monkey kidney (Vero) cells and porcine kidney 15 (PK-15) cells were kept in our lab and cultured in DMEM supplemented with 5% FBS at 37 °C in an incubator with 5% CO_2_. PEDV and TGEV strains were preserved in our laboratory and propagated in Vero and PK-15 cells, respectively.

### 2.4. Cell Viability Assay and Hemolysis Assay

The cytotoxicity of AgNPs on Vero and PK-15 cells was measured using the CCK-8 assay according to the manufacturer’s instructions. Briefly, the cells were seeded in a 96-well plate and cultured in a single layer for 24 h before treatment. The medium was removed, washed with phosphate-buffered saline (PBS) three times, treated with different concentration of AgNPs (2.5, 5, and 10 μg/mL) for 2 h and then washed with PBS and cultured for another 24 and 48 h. Then, the medium was replaced with DMEM containing 10 µL CCK-8 agent and incubated at 37 °C for 2 h. Absorbance of the samples was measured at 450 nm using a microplate reader.

Pure erythrocytes were collected from the blood of female BALB/c mice and prepared for hemolysis assay by gentle centrifugation at 3500 rpm at 4 °C and washing. Water and PBS was used as a positive or negative control, respectively. Samples were co-incubated at 37 °C for 4 h and centrifuged for photography, and the absorbance value of the supernatant at 540 nm was measured using a microplate detector.

### 2.5. Measurement of Viral Titer

The viral titer was measured using a median tissue culture infectious dose (TCID_50_) assay. Cells were cultured in 96-well plates to reach 80–90% confluence and infected with 10-fold serial dilutions of virus and infected for 1 h. The medium was replaced with DMEM maintained with 2% FBS and cultured for 3 days. The cytopathic effect (CPE) of the cells was observed, and the viral titer was calculated using the Reed–Muench method.

### 2.6. The Viricidal Effect of Chi-AgNPs on Virus

To measure the viricidal effects of AgNPs on coronaviruses, PEDV or TGEV with a multiplicity of infection (MOI) of 0.1 was mixed with AgNPs at 37 °C for 1 h, and virus mixtures were diluted 10-fold serially for viral TCID_50_ detection. The reduction in viral titers and the corresponding inhibition rate of viral infection were quantified and compared with those of the untreated virus.

### 2.7. One-Step Growth Curves

AgNPs and PEDV (MOI = 0.1) were premixed at 37 °C for 1 h, followed by infection with Vero cells for 1 h, and the virus mixtures was replaced with DMEM containing 2% FBS. Subsequently, the supernatants were collected at 12, 24, and 48 h post-infection (hpi) and used for viral titer measurements.

### 2.8. Indirect Immunofluorescence Assay

An indirect immunofluorescence assay (IFA) was used to further evaluate the inhibitory effect of AgNPs on PEDV infection. In brief, Vero cells at 80–90% confluence were cultured in a 24-well plate, and PEDV (MOI = 0.1) was pretreated with different concentrations of AgNPs (2.5, 5, and 10 μg/mL) before inoculation with cells. After 24 hpi, the cells were rinsed with PBST (containing 0.1% Tween 20) and fixed in cooled ethyl ethanol for 20 min at 4 °C, washed thrice with PBST, and permeabilized with 0.1% Triton X-100 at room temperature for 10 min. For immunofluorescence staining, cells were incubated with anti-PEDV S protein mouse polyclonal antibody (1:1000) for 2 h, washed with PBST, and subsequently incubated with FITC-labeled donkey anti-mouse IgG (1:2000) for 1 h. Finally, nuclei were stained with DAPI, and the slides were observed by fluorescence microscopy (Olympus BX-51, Olympus, Tokyo, Japan).

### 2.9. The Effect of Chi-AgNPs on Virus Attachment and Penetration

To investigate the effect of AgNPs on PEDV attachment, Vero cells were precooled at 4 °C incubated with PEDV (MOI = 0.1) cotreated with 10 μg/mL Chi-AgNPs at 4 °C for 1 h to allow virus attachment to cells. Subsequently, the cells were washed with PBS to remove the unbound virions, replaced with DMEM containing 2% FBS, and cultured at 37 °C. After 24 hpi, supernatants were collected for viral TCID_50_ detection, and cells were harvested with RIPA lysis buffer and used for Western blotting to analyze the protein expression level. For the viral penetration assay, Vero cells were precooled at 4 °C and further infected with PEDV (MOI = 0.1) at 4 °C for 1 h to allow virus attachment to cells but not entry. The virus was removed, washed with PBS, and incubated with Chi-AgNPs at 37 °C for 1 h to allow the virus to penetrate the cells. Afterward, cells were washed with PBS and cultured at 37 °C for 24 h. The supernatants and cells were harvested for viral TCID_50_ detection and Western blot analysis, respectively.

### 2.10. Western Blot

Cells were harvested, washed twice with PBS, and incubated with RIPA lysis buffer containing 1 mM phenylmethyl sulfonylfluoride (PMSF) at 4 °C for 10 min. Protein concentrations were determined using BCA protein assay kits. The lysed samples were subjected to 10% sodium dodecyl sulfateepolyacrylamide gel electrophoresis (SDS-PAGE) and transferred to a polyvinylidene difluoride (PVDF) membrane using a protein transfer device (Bio-Rad, Hercules, CA, USA) at 70 V for 90 min. Thereafter, the membrane was blocked with 5% non-fat dry milk in PBST at 4 °C overnight and incubated with anti-PEDV N or S protein mouse polyclonal antibody (1:1000) for 2 h, which was followed by HRP-labeled goat anti-mouse IgG (1:3000) for 1 h. Signals were visualized with an enhanced Western blot substrate using a chemiluminescence instrument (Bio-Rad, Hercules, CA, USA). Relative protein expression levels were analyzed using Image J software v1.8.0.

### 2.11. Determination of ROS Production

Cellular ROS production was determined using a ROS detection kit. Vero cells were seeded in a 24-well plate and infected with different MOI of PEDV (MOI = 0.1, 0.2, and 0.4) and then incubated for 12, 24, and 48 h. Cellular ROS levels in Vero cells were infected with PEDV (MOI = 0.2) pretreated with different concentrations of Chi-AgNPs (2.5, 5, and 10 μg/mL) for 1 h and then detected after 48 hpi. After removing the medium, cells were stained with 10 μM 2′,7′-dichloroflfluorescein diacetate (DCFH-DA) at 37 °C for 20 min in the dark, and the ROS levels were detected by DHE fluorescence intensity using fluorescence microscopy.

### 2.12. Detection of Mitochondrial Membrane Potential

The change in mitochondrial membrane potential (MMP) was measured by a JC-1 kit. Chi-AgNPs (5 μg/mL) were treated with PEDV (MOI = 0.1) at 37 °C for 1 h, and the virus mixtures were added to Vero cells for 1 h, which was followed by washing with PBS and incubation for 24 h. The subsequent steps were conducted according to the instructions of the JC-1 kit, and 10 μM CCCP provided in the kit was used as the positive control. Fluorescent images of JC-1 monomers and aggregates were observed using a fluorescence microscope.

### 2.13. Flow Cytometry Analysis of Cell Apoptosis

Cell apoptosis induced by PEDV was analyzed by flow cytometry using an Annexin V-FITC and a propidium iodide (PI) dual fluorescent dye kit, according to the manufacturer’s instructions. Vero cells were seeded in a 6-well plate and incubated with PEDV (MOI = 0.1) pretreated with Chi-AgNPs (5 μg/mL) for 1 h, infected for 1 h, followed by washing with PBS, and cultured for 24 h. The cells were trypsinized and centrifuged at 1000 rpm for 5 min, then re-suspended in 100 µL of binding buffer, incubated with 5 µL of Annexin V-FITC and 10 µL of PI for 10 min in the dark, and then 400 µL of binding buffer was added. Finally, the percentage of stained cells was analyzed using flow cytometry (CytoFLEX, Beckman, CA, USA).

### 2.14. TEM Analysis of the Interaction between PEDV and Chi-AgNPs

PEDV suspension mixtures (1.0 × 10^7^ TCID_50_, 150 μL) and Chi-AgNPs (200 μg/mL, 50 μL) were incubated at room temperature for 30 min, and then virus mixtures were adsorbed onto carbon-coated copper grids for 10 min, which was followed by staining with 1% phosphotungstic acid negative stain solution for 5 min. Subsequently, the samples were observed using TEM (JEOL JEM-2100, Tokyo, Japan).

### 2.15. Ellman’s Assay

Ellman’s assay was performed according to the manufacturer’s instructions. Briefly, 50 μL of Chi-AgNPs was mixed with 50 μL of the PEDV suspension at room temperature for 60 min, and then it was centrifuged at 14,000 rpm for 30 min to remove AgNPs from the mixtures. In parallel, 10 mM TCEP was reacted with 100 μL of PEDV suspension at room temperature for 60 min as a positive control. Subsequently, the DTNB solution was added to the mixture, and the absorbance of the solution at 412 nm was measured using a microplate reader.

### 2.16. Analysis of Secondary Structure by Circular Dichroism

The secondary structure of the PEDV S protein in the presence or absence of Chi-AgNPs was analyzed using circular dichroism (CD, J-810, Jasco, Tokyo, Japan). The S protein (200 μg/mL in PBS) was incubated with a final concentration of 10 μg/mL Chi-AgNPs at room temperature for 60 min and then centrifuged at 14,000 rpm for 30 min to remove AgNPs in the mixtures. S protein treated with a final concentration of 10 mM TCEP was used as a positive control. CD spectra were recorded from 190 to 260 nm. The spectral results were analyzed using the CDNN software to calculate the percentage of secondary structures.

### 2.17. Statistical Analysis

The data were shown as mean ± standard deviation (SD). One-way ANOVA and *t*-test were used to analyze the differences between groups. The * *p* < 0.05, ** *p* < 0.01, and *** *p* < 0.001 were considered statistically significant.

## 3. Results and Discussion

### 3.1. Characterization of Chi-AgNPs

**Figure 1:**
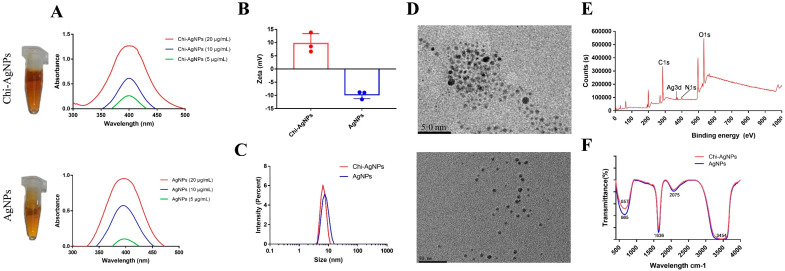
Characterization of Chi-AgNPs and AgNPs. (**A**) UV−visible absorption spectra of the Chi-AgNPs and AgNPs. (**B**) Zeta potentials of Chi-AgNPs and AgNPs. (**C**) Size distribution of Chi-AgNPs and AgNPs measured by DLS. (**D**) The TEM images of Chi-AgNPs and AgNPs. (**E**) The XPS of AgNPs. (**F**) The FTIR spectrum of AgNPs.

Two types of AgNPs with positive and negative charges were successfully prepared and characterized using UV-vis spectroscopy, dynamic light scattering (DLS), TEM, FT-IR spectra, and XPS. The UV-vis absorption spectra of Chi-AgNPs and AgNPs with a characteristic silver plasmon band at 400 nm are shown in Figure 1A. The morphologies of the Chi-AgNPs and AgNPs were observed via TEM, and their size distributions were determined via DLS. As shown in Figure 1B, the positive charge of Chi-AgNPs (9.65 ± 3.72 mV) and DLS measurements showed that the sizes of Chi-AgNPs and AgNPs were approximately 7 nm and 8 nm, respectively (Figure 1C). The negative charge of AgNPs (−9.76 ± 1.51 mV) was observed. TEM images showed spherical particles (Figure 1D). Elemental analysis by XPS showed four peak spectra for AgNPs at 284, 367, 399, and 531 eV, which were attributed to C, Ag, N, and O elements, respectively (Figure 1E). The FT-IR spectra of the Chi-AgNPs exhibited an absorption peak at 3454 cm^−1^ owing to the tensile vibration of the O-H bond. The absorption peaks at 2075 and 1636 cm^−1^ were attributed to C-C and C=C bonds stretching (Figure 1F), and the results of the FT-IR spectra were consistent with those of previous studies [2,22].

### 3.2. Biocompatibility Analysis of Chi-AgNPs

**Figure 2:**
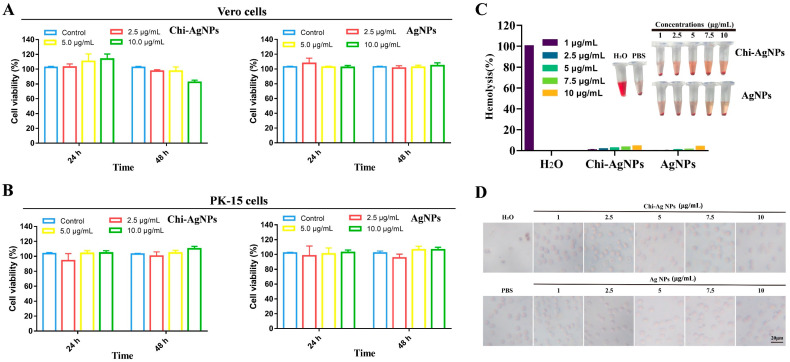
Biocompatibility analysis of Chi-AgNPs. The cytotoxicity of Chi-AgNPs and AgNPs on Vero and PK-15 cells were determined by CCK-8 assay. Vero cells (**A**) or PK-15 cells (**B**) were pretreated with the indicated doses of Chi-AgNPs or AgNPs for 2 h and then washed with PBS and cultured for another 24 and 48 h. Cell viability was measured by CCK-8 assay. (**C**) Hemolysis analysis of various concentrations of Chi-AgNPs and AgNPs in vitro, while ddH_2_O and PBS was used as a positive or negative control, respectively. (**D**) Morphology of erythrocytes that co-incubated with different concentrations of samples. The error bars indicate means ± SD (*n* = 3).

We first investigated the cytotoxicity of Chi-AgNPs in Vero and PK-15 cells, which is a type of PEDV or TGEV infected cell line in vitro. The cytotoxic effects of Chi-AgNPs on Vero and PK-15 cells were determined using a CCK-8 assay. The viability of the two kinds of cell types incubated with Chi-AgNPs and AgNPs were determined, respectively. As shown in Figure 2A,B, after incubation for 24 and 48 h, no cytotoxic effect was observed in Vero and PK-15 cells treated with 2.5, 5, and 10 μg/mL of the two AgNPs compared to the untreated group, and cell morphology was not affected at this concentration. Thus, concentrations ranging from 2.5 to 10 μg/mL were used for further testing. The results showed that no obvious cytotoxicity was detected in Vero and PK-15 cells treated with the two AgNPs (10 μg/mL), which is consistent with previous reports [23,24].

The biocompatibility of Chi-AgNPs was evaluated by an in vitro hemolysis test using blood. The hemolysis rate of the Chi-AgNPs and AgNPs group was lower than 2.5% at 5 μg/mL, while the hemolysis rate of the water group was 100% (Figure 2C). The Chi-AgNPs and AgNPs group was morphologically intact, similar to the PBS group, demonstrating excellent biocompatibility properties (Figure 2D). These results suggested that Chi-AgNPs have good biocompatibility in vitro, further providing a promising nanomaterial for their antiviral applicability.

### 3.3. Chi-AgNPs Antiviral Effect by Directly Targeting Virions

**Figure 3:**
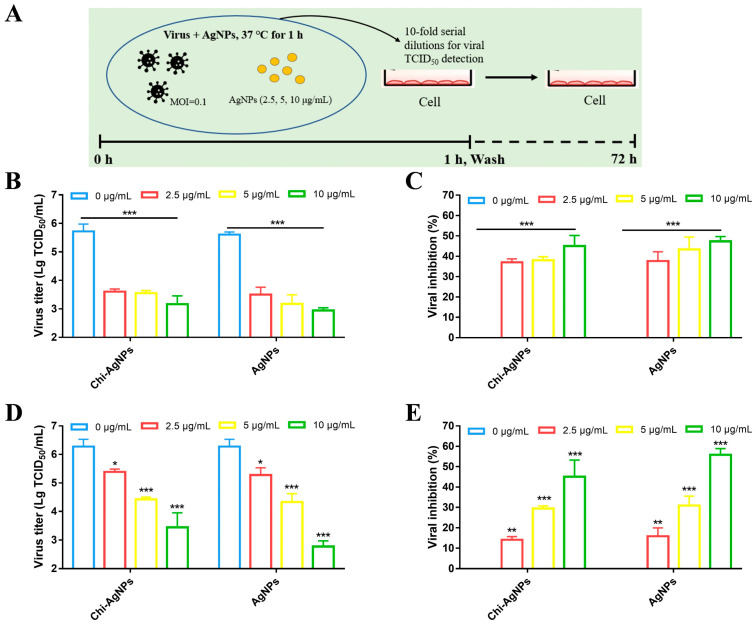
Chi-AgNPs antiviral effect by directly targeting virions. (**A**) Schematic representation of the viricidal effect of Chi-AgNPs on virus. PEDV (**B**) or TGEV (**D**) were incubated with Chi-AgNPs (2.5, 5, 10 μg/mL) at 37 °C for 1 h, and then the virus mixtures were diluted 10-fold serially for viral TCID_50_ detection. The corresponding inhibition rate of PEDV (**C**) or TGEV (**E**) infection was quantified. The error bars indicate means ± SD (*n* = 3), * *p* < 0.05, ** *p* < 0.01, *** *p* < 0.001.

PEDV and TGEV were selected to explore the antiviral effects of Chi-AgNPs on swine coronaviruses. To investigate the viricidal effect on virions, PEDV or TGEV was directly incubated with AgNPs (2.5, 5, and 10 μg/mL) at 37 °C for 1 h. Then, the virus mixtures were serially diluted 10-fold, and viral titers were determined using a TCID_50_ assay at 24 hpi (Figure 3A). For comparison, the antiviral effects of the Chi-AgNPs and AgNPs were tested under similar experimental conditions. As shown in Figure 3B, compared with the untreated group, direct AgNPs treatment with various concentrations (2.5, 5, and 10 μg/mL), the PEDV titer reduced by 2.11, 2.17, and 2.55 log TCID_50_ in the Chi-AgNPs group, while it was reduced by 2.11, 2.43, and 2.67 log TCID_50_ in the AgNPs group. The corresponding inhibition rates of PEDV infection are quantified in Figure 3C. It was found that AgNPs impaired virus entry with approximately 48% efficiency at a concentration of 10 μg/mL. The PEDV titer showed a 100-fold decrease at 2.5 μg/mL of Chi-AgNPs higher than that of the reported Au@Ag nanorods (Au@AgNRs) [2]. Similarly, Chi-AgNPs significantly reduced the TGEV titer with a dose-dependent manner. The TGEV titer reduced by 0.88, 1.85, and 2.83 log TCID_50_ in the Chi-AgNPs group, respectively, while it was reduced by 0.99, 1.94, and 3.5 log TCID_50_ in the AgNPs group, respectively (Figure 3D,E). This study indicated that Chi-AgNPs reduced the viral titer by at least 10-fold higher than that of the previously reported antiviral compound [25], demonstrating that Chi-AgNPs exhibited a more potent viricidal effect on CoVs.

### 3.4. The Inhibitory Effect of Chi-AgNPs on Virus Replication

**Figure 4:**
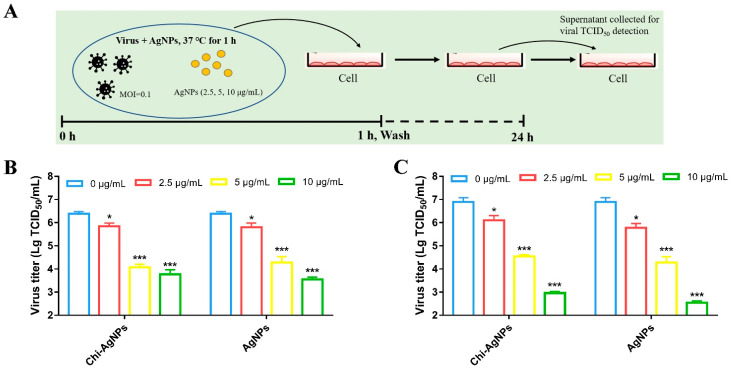
The inhibitory effect of Chi-AgNPs on virus replication. (**A**) Schematic representation of the inhibitory effect of Chi-AgNPs on virus replication. Chi-AgNPs and PEDV (**B**) or TGEV (**C**) (MOI = 0.1) were pretreated at 37 °C for 1 h in vitro and then incubated with Vero or PK-15 cells for 1 h at 37 °C, respectively. Next, the cells washed with PBS and cultured for another 24 h, and the supernatant was collected for viral TCID_50_ detection. The error bars indicate means ± SD (*n* = 3), * *p* < 0.05, *** *p* < 0.001.

Next, we evaluated the inhibitory effects of the Chi-AgNPs on viral replication. The effect of Chi-AgNPs on viral replication was determined by measuring the viral titer in the presence of various concentrations of AgNPs at 24 hpi (Figure 4A). As expected, treatment with Chi-AgNPs strongly inhibited the replication of PEDV and TGEV. The viral titer was significantly reduced in PEDV-infected Vero cells (Figure 4B) and TGEV-infected PK-15 cells (Figure 4C) treated with various concentrations of Chi-AgNPs or AgNPs, indicating that Chi-AgNPs exhibited a strong inhibitory effect on viral replication. These results showed that the viral titer was significantly reduced in the presence of the two types of AgNPs in a dose-dependent manner. Additionally, they exhibited similar antiviral activity by directly targeting virions, implying that there were no charge-dependent differences in the zeta potential of the modified AgNPs on viricidal effects on PEDV and TGEV.

### 3.5. Chi-AgNPs Exhibit Antiviral Activity of PEDV

**Figure 5:**
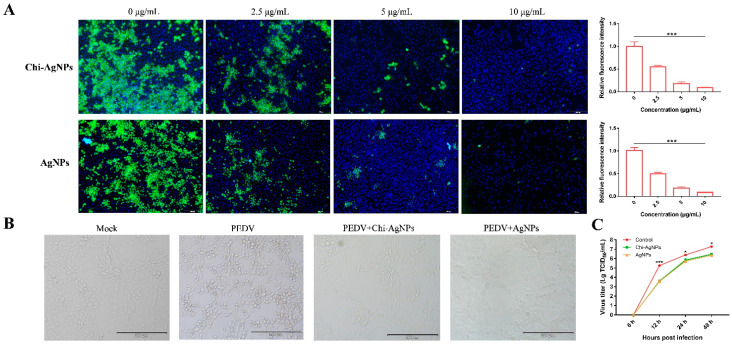
Chi-AgNPs exhibit antiviral activity against PEDV. (**A**) Indirect immunofluorescence assay of PEDV-infected Vero cells (MOI = 0.1) treated with different concentrations of Chi-AgNPs or AgNPs (2.5, 5, 10 μg/mL) at 24 hpi (Scale bar = 200 μm). Anti-PEDV spike (S) protein mouse polyclonal antibody (1:1000). The relative fluorescence intensity of PEDV S at 24 hpi was analyzed by ImageJ. (**B**) The cytopathic effect (CPE) result of Vero cells infected with untreated and Chi-AgNPs-treated PEDV. The mock Vero cells and infected with AgNPs-treated PEDV exhibited no CPE. An obvious CPE is observed in PEDV-infected Vero cells (Scale bar = 200 μm). (**C**) Growth curves of PEDV treated or untreated with Chi-AgNPs and AgNPs (2.5 µg/mL). PEDV-infected Vero cells were cultured for an indicated time period (12, 24, and 48 h). The error bars indicate means ± SD (*n* = 3), * *p* < 0.05, *** *p* < 0.001.

An immunofluorescence assay (IFA) was used to evaluate the effect of Chi-AgNPs on PEDV proliferation in Vero cells by detecting S protein expression levels. Polyclonal antibodies specific to PEDV S protein and FITC-labeled donkey anti-mouse IgG were used to analyze the amount of PEDV in Vero cells. The cell nucleus and PEDV S protein emitted blue and green fluorescence, respectively. As shown in Figure 5A, compared with PEDV-infected cells, both Chi-AgNPs and AgNPs-treated cells showed a significant decrease in green fluorescence, indicating a significant inhibition of S protein and virus proliferation in Vero cells. Furthermore, the inhibitory effect of Chi-AgNPs on PEDV proliferation was examined by observing the morphology of the PEDV-infected cells. PEDV was pretreated with Chi-AgNPs for 1 h prior to infection, and images of cytopathic effects (CPEs) were obtained to observe the impact on virus infectivity. As shown in Figure 5B, an obvious CPE was found in PEDV-infected cells but not in mock Vero cells and Chi-AgNPs-treated PEDV infected cells, demonstrating that Chi-AgNPs might impair the infection ability of PEDV by interacting directly with the viral envelope. Moreover, viral titer detection of PEDV at 12, 24, and 48 hpi demonstrated significant inhibition of the one-step growth curve of PEDV after Chi-AgNPs treatment (Figure 5C), revealing the potent role of Chi-AgNPs in blocking PEDV infection.

### 3.6. Chi-AgNPs Inhibit PEDV Infection at the Attachment and Penetration Step

**Figure 6:**
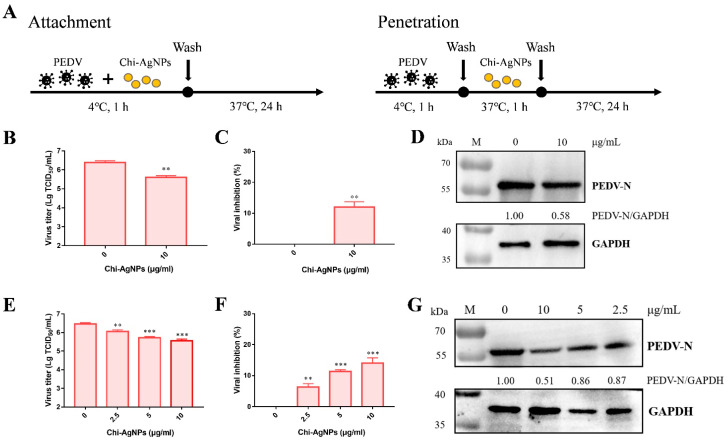
Chi-AgNPs inhibit PEDV attachment and penetration. (**A**) Schematics of PEDV attachment and penetration assay. For virus attachment assay, Vero cells were precooled at 4 °C, which was followed by PEDV (MOI = 0.1) incubation in the presence of Chi-AgNPs at 4 °C for another 1 h. Then, cells were washed with PBS three times and cultured at 37 °C for 24 h. (**B**,**C**) Supernatants were collected for viral titers and viral inhibition measurement by a TCID_50_ assay. (**D**) The corresponding expression of PEDV N protein was determined by Western blot and quantified by ImageJ. Chi-AgNPs block PEDV penetration. Vero cells were precooled at 4 °C, which was followed by PEDV (MOI = 0.1) incubation for 1 h. Then, cells were washed with PBS three times and incubated in the presence of Chi-AgNPs at 37 °C for 1 h. (**E**,**F**) At 24 hpi, supernatants were collected for viral titers and viral inhibition measurement by a TCID_50_ assay. (**G**) Cells were harvested for PEDV N protein expression analysis by Western blot. The error bars indicate means ± SD (*n* = 3), ** *p* < 0.01, *** *p* < 0.001. Original images of (**D**,**G**) can be found in Supplementary Materials.

Although the two types of AgNPs showed strong antiviral abilities, chitosan-modified carbohydrate polymers have been widely used for biological applications due to specific characteristics, such as good biodegradability, biocompatibility, and versatility, particularly their safe treatment capability [26,27]. In addition, chitosan-modified metal–organic nanomaterials can improve their activity and stability, making them ideal tools for antimicrobial applications [28]. Based on these superior properties, Chi-AgNPs were selected for subsequent experiments to elucidate their potential antiviral mechanism. The interaction between Chi-AgNPs and the virus is the major stage of infection by blocking virus entry and disrupting virus replication and budding. To understand the disturbance stage of Chi-AgNPs during the viral life cycle, viral attachment and penetration assays were performed (Figure 6A). First, PEDV was incubated with Vero cells in the existence of Chi-AgNPs at 4 °C for 1 h, and then the cells were washed with PBS three times and incubated at 37 °C for 24 h. Figure 6B indicates that the treatment of PEDV with 10 μg/mL Chi-AgNPs reduced viral attachment to cells by 12%. To investigate the effect of Chi-AgNPs on penetration, PEDV was allowed to attach to Vero cells at 4 °C for 1 h and then penetrate the cells in the presence of Chi-AgNPs at 37 °C for 1 h. As expected, the inhibition rate of PEDV incubated with Chi-AgNPs was decreased by approximately 6–14%. Meanwhile, Western blot analysis showed a downregulation of the PEDV N protein after treatment with Chi-AgNPs (Figure 6G). These data support the function of Chi-AgNPs in inhibiting PEDV attachment and penetration, ultimately inhibiting viral infection.

### 3.7. The Interaction Mechanism between Chi-AgNPs and PEDV

**Figure 7:**
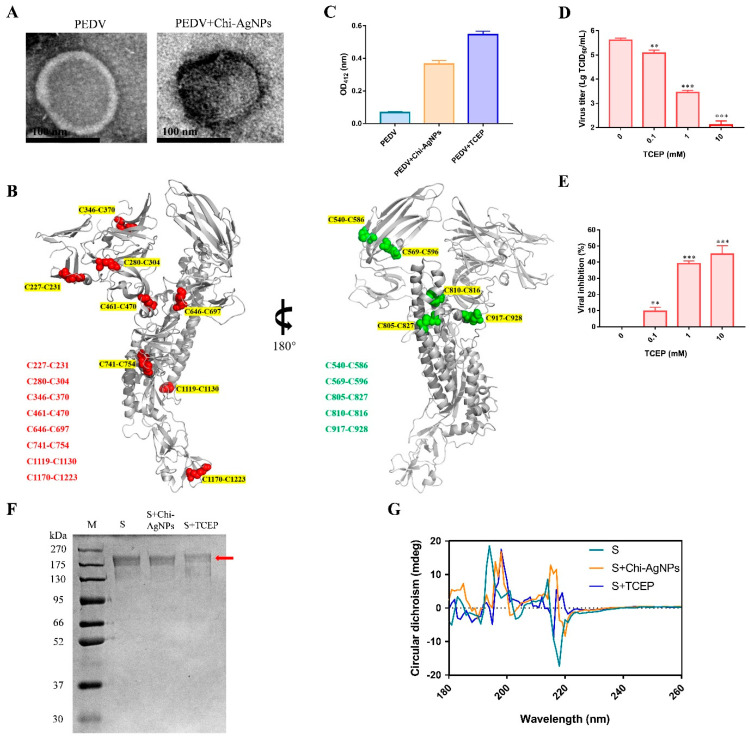
The interaction mechanism between Chi-AgNPs and PEDV. (**A**) TEM image of PEDV and Chi-AgNPs−treated PEDV. (**B**) The disulfide bridges based on resolved PEDV S protein crystal structure and the cysteine residues are shown in red and green. TCEP inhibits the infectivity of PEDV. (**C**) Ellman’s assay conducted with PEDV suspension that interacted with Chi-AgNPs for 60 min. (**D**) PEDV was incubated with TCEP (0.1, 1, 10 mM) at 37 °C for 1 h, and then the virus mixtures were diluted 10-fold serially for viral TCID_50_ detection. (**E**) The corresponding inhibition rate of PEDV infection was quantified. (**F**) SDS-PAGE analysis of PEDV S protein. The red arrow indicates the S protein band. M: protein marker; S: S protein without any treatment as control; S + Chi-AgNPs: S protein treated with 10 μg/mL Chi-AgNPs for 1 h; S + TCEP: S protein treated with a final concentration of 10 mM TCEP. (**G**) Circular dichroism (CD) spectra of S protein. The spectra were measured from 180 to 260 nm. The error bars indicate means ± SD (*n* = 3), ** *p* < 0.01, *** *p* < 0.001.

Viral entry depends on strong interactions between virions and host cells [29]. Chi-AgNPs have been reported to block viral entry into host cells via direct interaction with viral structural proteins, destroying the viral envelope, and blocking viral receptor binding sites [15,30]. In this study, TEM analysis was performed to determine the interaction between Chi-AgNPs and virions. Briefly, the PEDV suspension was incubated with Chi-AgNPs at a final concentration of 50 μg/mL at room temperature for 30 min, and then the virus mixtures were used for TEM observation. Figure 7A shows that a viral envelope with a diameter of approximately 10 nm was clearly observed in the TEM image, while Chi-AgNPs treatment caused an envelope destruction of virions. As enveloped viruses need to trigger the membrane fusion pathway via fusing their envelope with the host cell membrane to release the viral genome into the cells, viral envelope destruction reduces viral entry.

Structural proteins maintaining the viral morphology are responsible for cell entry. PEDV can directly attach to the cell surface after binding to specific receptors, followed with internalization via viral surface structural proteins-mediated endocytosis and subsequent entry into host cells. Among these proteins, S protein is a homo-trimeric surface glycoprotein, which can facilitate viral membrane fusion and internalization via promoting viral attachment to host cell receptors [29,31,32]. For CoVs, the disulfide bonds in the S protein can affect viral infection by obstructing viral attachment [33]. The crystal structure of the PEDV S protein demonstrated 13 disulfide bridges, which are possible for disulfide bond formation, and the cysteine residues are shown in red and green (Figure 7B). Notably, 7 of the 13 disulfide bridges were observed in the α helix to solidify the S protein structure. Viral infection was inhibited by blocking viral attachment. Consequently, we investigated the importance of disulfide bonds in the S protein. Thus, we hypothesized that the inactivation of PEDV caused by cleavage of the disulfide bonds is required for viral membrane fusion to its host cells. To verify this hypothesis, TCEP was selected as a disulfide bond reducing agent to test the roles of disulfide bonds in PEDV infection. Ellman’s assay (DTNB reduction assay) was performed to detect the reduced disulfide bonds in the viral spike protein that interacted with Chi-AgNPs. As shown in Figure 7C, the absorbance at 412 nm of Chi-AgNPs or TCEP-treated PEDV was higher than that of the control group, indicating that a higher sulfhydryl group concentration in the virus as well as a great reduction in disulfide bonds exists, which provides evidence for supporting the relationship between viral disulfide bond reduction and attenuated virus infectivity. As expected, Figure 7D shows that TCEP-treated PEDV significantly lost their infection ability in Vero cells, as the titer of PEDV treated with 0.1, 1, and 10 mM TCEP was reduced by 0.54, 2.17, and 3.51 log TCID_50_, respectively, suggesting that the disulfide bond is necessary for the fusion between virus and cells. Using the purified PEDV S protein, we analyzed the effects of Chi-AgNPs on the secondary structure of the S protein. Meanwhile, TCEP was used as the positive control. As shown in Figure 7F, the S protein that was treated with Chi-AgNPs and TCEP was verified by SDS-PAGE. Furthermore, circular dichroism (CD) spectroscopy assay indicated that the Chi-AgNPs and TCEP-treated groups dramatically changed the circular dichroism (CD) spectra of the S protein from 180 to 220 nm (Figure 7G), which caused a decrease in the α helix from 11.8% to 7.8% or 6.4% and an increase in β-sheet from 38.0% to 48.5% or 53.4%, respectively (Table 1). These results suggested that Chi-AgNPs destroyed the disulfide bonds that affected the S protein secondary structure by decreasing its α helix content, which was similar to that of TCEP-treated virions that cleave the disulfide bonds in the S protein, which reduces PEDV infection.

The virucidal activity of AgNPs depends on the shape, size, concentration, and sensitivity of the virus [15]. The mechanisms of action of metal nanoparticles that exhibit antiviral activity include the disruption of disulfide bonds that maintain the viral particle structure or release of metal ions that interact with viral envelopes. Kim et al. reported that the higher relative surface area of AgNPs with higher affinity to the disulfide bonds in influenza virus hemagglutinin (HA) caused a big difference in disulfide bond reduction [16]. Furthermore, metal nanoparticles can activate the host antiviral immune response and facilitate the phagocytosis by macrophages for efficiently virus elimination by the host, which would be beneficial for therapeutic applications in vivo.

### 3.8. Chi-AgNPs Reduce PEDV-Induced Apoptosis by Inhibiting ROS Production

**Figure 8:**
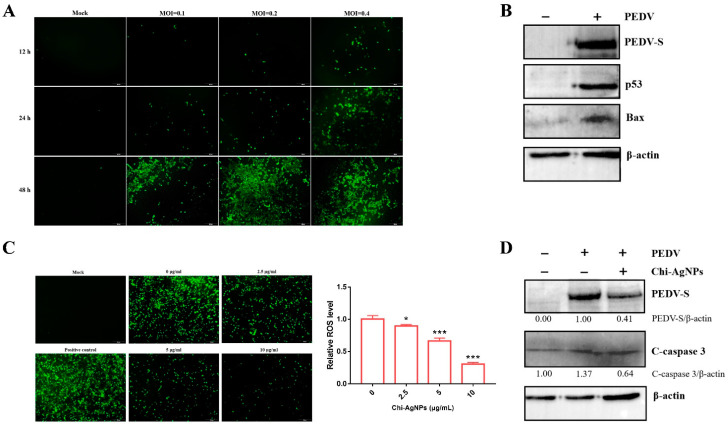
Chi-AgNPs reduce PEDV-induced apoptosis by inhibiting ROS generation. (**A**) Cellular ROS levels in Vero cells at different PEDV infection time points. ROS levels were detected by DHE fluorescence intensity (Scale bar = 100 μm). (**B**) PEDV infection induces p53 signaling activation. Western blot analysis of PEDV S protein, p53, and Bax in Vero cells infected with PEDV. (**C**) ROS values significantly decreased in the Chi-AgNPs-treated cells compared to the untreated group (Scale bar = 100 μm). The corresponding statistical histogram showing the relative ROS level. (**D**) Chi-AgNPs (5 μg/mL) inhibit cell apoptosis during PEDV infection. Western blot analysis of the expression of cleaved caspase 3 and PEDV S protein level in PEDV infected Vero cells treated with Chi-AgNPs. The error bars indicate means ± SD (*n* = 3), * *p* < 0.05, *** *p* < 0.001. Original images of (**B**,**D**) can be found in Supplementary Materials.

PEDV infection has been suggested to induce oxidative stress in cells by generating ROS [34,35], as excess cellular levels of reactive oxygen species (ROS) lead to protein damage, revealing an essential role in cell apoptosis [36]. In this study, fluorescence imaging based on a DCF probe was performed in Vero cells infected with different MOI of PEDV to determine ROS levels. As shown in Figure 8A, ROS levels gradually increased with increasing infection time in PEDV-infected cells. Western blotting was used to analyze the levels of p53, a major transcription factor for the manipulation of apoptosis in PEDV-infected Vero cells, which was considered to be responsible for the upregulation of the Bax expression level. Consistent with previous reports [37], the levels of p53 and Bax were higher in PEDV-infected Vero cells than in mock cells (Figure 8B), indicating that PEDV infection induces cell apoptosis through p53 signaling activation. As mentioned above, these data show that PEDV promotes ROS production and p53 activation to induce cell apoptosis. Next, we investigated the effect of Chi-AgNPs on the PEDV-induced apoptosis. As expected, the results showed that the levels of ROS in Chi-AgNPs treatment significantly reduced in a dose-dependent manner (Figure 8C). The PEDV S protein and cleaved caspase 3 levels in Vero cells decreased significantly after Chi-AgNPs treatment (Figure 8D). These results showed that Chi-AgNPs reduced PEDV-induced apoptosis by inhibiting ROS production.

### 3.9. Chi-AgNPs Inhibit Cell Apoptosis during PEDV Infection via Regulating p53-Mediated Apoptotic Pathway

**Figure 9:**
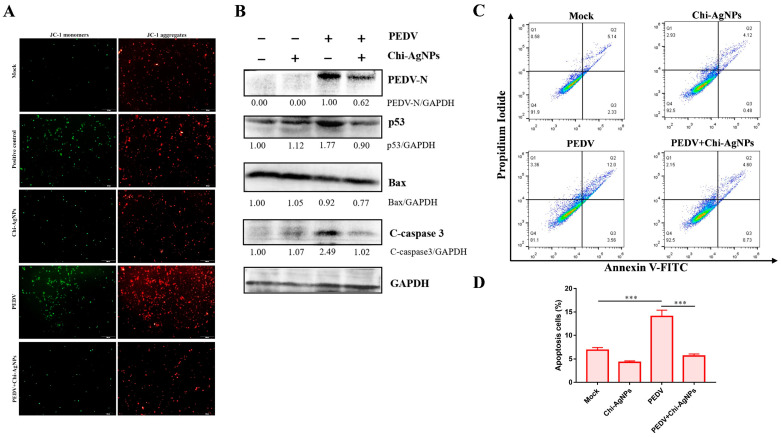
Chi-AgNPs inhibit cell apoptosis during PEDV infection via regulating p53-mediated apoptotic pathway. (**A**) Mitochondria membrane potential of PEDV infection in Vero cells treated with Chi-AgNPs (5 μg/mL). Cells treated with CCCP provided in JC-1 kit used as a positive control. After staining, JC-1 monomers and aggregates show green and red fluorescence, respectively (Scale bar = 200 μm). (**B**) Chi-AgNPs (5 μg/mL) inhibit PEDV-induced cell apoptosis through p53-mediated apoptotic pathway. Western blot analysis of the expression of PEDV N protein, p53, Bax, and cleaved caspase 3. (**C**,**D**) The apoptosis rates were analyzed by flow cytometry. The error bars indicate means ± SD (*n* = 3), *** *p* < 0.001. Original images of (**B**) can be found in Supplementary Materials.

The decrease of mitochondrial membrane potential (MMP) can lead to the destruction of mitochondria, accompanied by the release of several regulatory factors including caspase 3 protein into the cytoplasm [38]. Next, the JC-1 kit was used to analyze the effect of Chi-AgNPs on the MMP of PEDV-infected Vero cells. As shown in Figure 9A, JC-1 monomers with strong green fluorescence were observed in PEDV-infected Vero cells and CCCP-treated cells (positive control) due to the destruction of mitochondrial integrity by PEDV infection, which finally resulted in a decrease in MMP. However, the presence of Chi-AgNPs dramatically reversed the decrease in green fluorescence, the intensity of which was similar to that of mock and only Chi-AgNPs-treated cells. This result demonstrated that Chi-AgNPs could protect the destruction of mitochondrial integrity caused by PEDV. Consistent with this result, Chi-AgNPs treatment dramatically reversed the PEDV-induced upregulation of p53, Bax, and cleaved caspase 3 induced by PEDV (Figure 9B). In addition, FACS assay indicated that the apoptosis rate of Vero cells infecting with PEDV was 14.1%. In contrast, the apoptosis ratio of Chi-AgNPs-treated PEDV was only 5.6%, which was even lower than that of the mock cells (6.8%) (Figure 9C,D), indicating the inhibitory effect of Chi-AgNPs against PEDV-induced cell apoptosis. Collectively, these results suggest that Chi-AgNPs inhibit PEDV infection and the apoptosis of Vero cells through activation of the p53-mediated signaling pathway.

## 4. Conclusions

In this study, two types of AgNPs (Chi-AgNPs and AgNPs) with different charges were prepared to investigate their antiviral effects on swine CoVs. The two AgNPs showed similar antiviral effects on CoVs, suggesting no charge-dependent manner of the modified AgNPs on the viricidal effect on CoVs. The antiviral effects of Chi-AgNPs demonstrated that they prevented viral infection by inhibiting PEDV attachment and penetration. In addition, the direct interaction between Chi-AgNPs and PEDV surface can destroy the viral S protein structure, subsequently blocking virus binding to cell receptors. These key processes associated with membrane fusion were caused by the cleavage of disulfide bond in the S protein. Moreover, the inhibitory effects of Chi-AgNPs on PEDV induced cell apoptosis via ROS/p53 signaling activation in Vero cells. Our findings elucidate the interaction mechanism of the antiviral effect of Chi-AgNPs on CoVs.

## Figures and Tables

**Table 1:** Percentage of the secondary structure of S protein.

Treatment	α Helix	β-Sheet	Beta-Turn	Rndm. Coil
S	11.8%	38.0%	19.0%	31.2%
S + Chi-AgNPs	7.8%	48.5%	17.2%	26.5%
S + TCEP	6.4%	53.4%	13.9%	26.3%

## Data Availability

Data available on request.

## Supplementary Materials

The following supporting information can be downloaded at: https://www.mdpi.com/article/10.3390/biom14091152/s1, Original images of Figure 6D,G, Figure 8B,D and Figure 9B can be found in Supplementary Materials.
