# Supplementary material for: Chitosan-Modified AgNPs Efficiently Inhibit Swine Coronavirus-Induced Host Cell Infections via Targeting the Spike Protein
Source: Biomolecules. 2024 Sep 13;14(9):1152. doi: 10.3390/biom14091152 (PMC11430280; doi:10.3390/biom14091152)
Supplement: Supplementary file 1 [file biomolecules-14-01152-s001.zip › Original images.pdf]

Original images

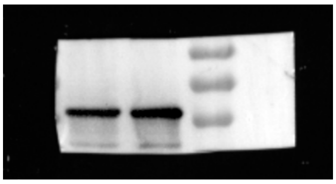

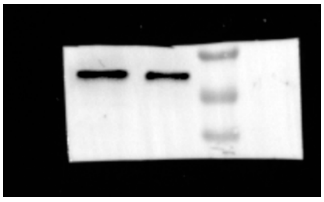

Edited images

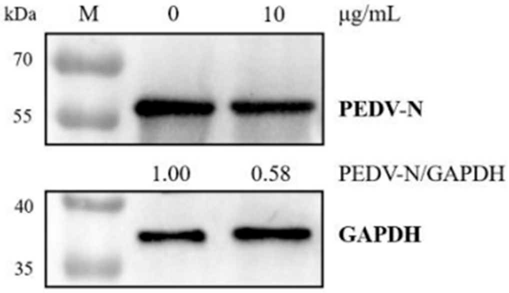

Fig. 6D The corresponding expression of PEDV N protein was determined by western blot and quantified by ImageJ. Chi-AgNPs blocks PEDV penetration. Vero cells were pre-cooled at 4 °C, followed by PEDV (MOI=0.1) incubation for 1 h. Then, cells were washed with PBS three times and incubated in the presence of Chi-AgNPs at 37 °C for 1 h.

Original images

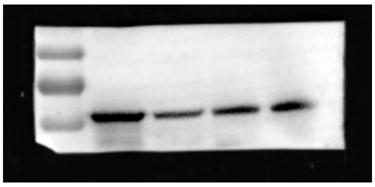

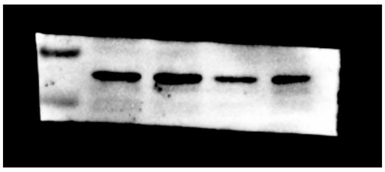

Edited images

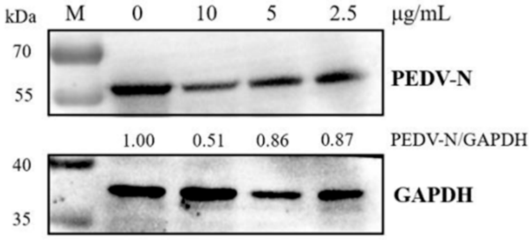

Fig. 6G Cells were harvested for PEDV N protein expression analysis by western blot.

Original images

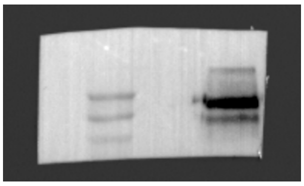

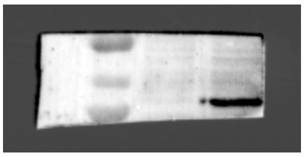

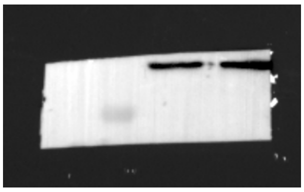

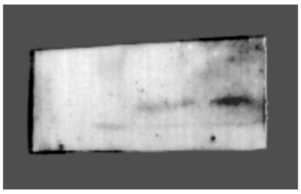

Edited images

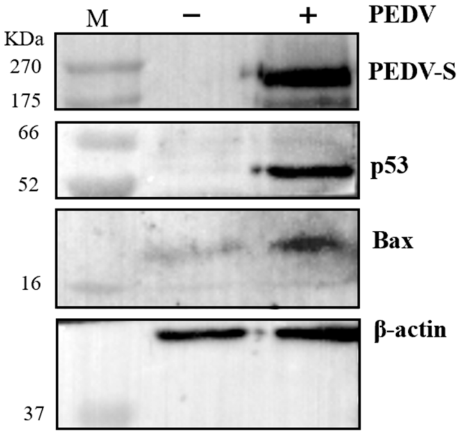

Fig. 8B PEDV infection induces p53 signaling activation. Western blot analysis of PEDV S protein, p53, and Bax in Vero cells infected with PEDV.

Original images

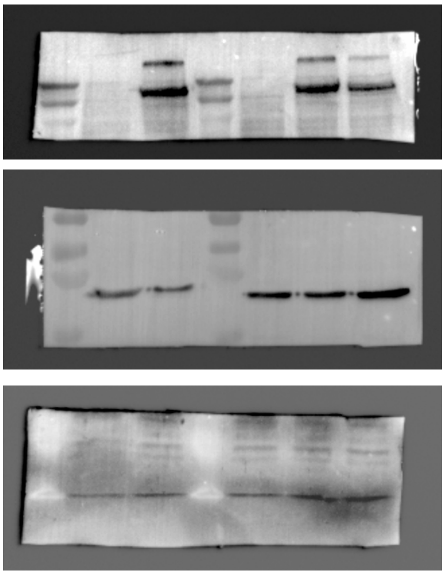

Edited images

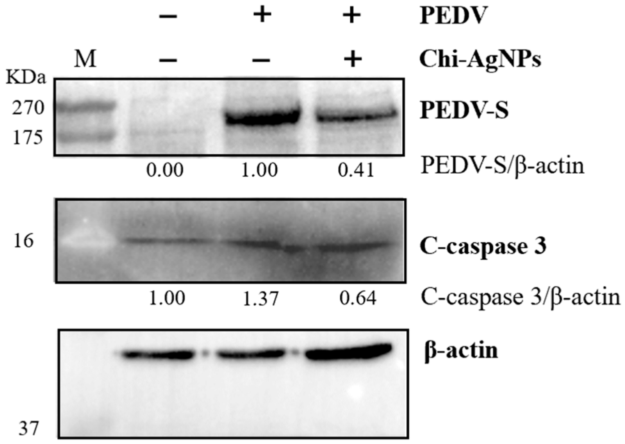

Fig. 8D Chi-AgNPs inhibit cell apoptosis during PEDV infection. Western blot analysis of the expression of cleaved caspase 3 and PEDV S protein level in PEDV infected Vero cells treated with Chi-AgNPs.

Original images

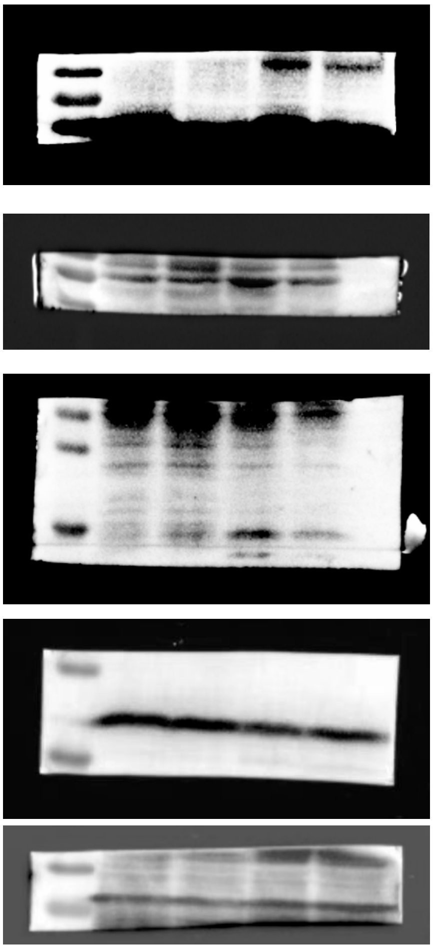

Edited images

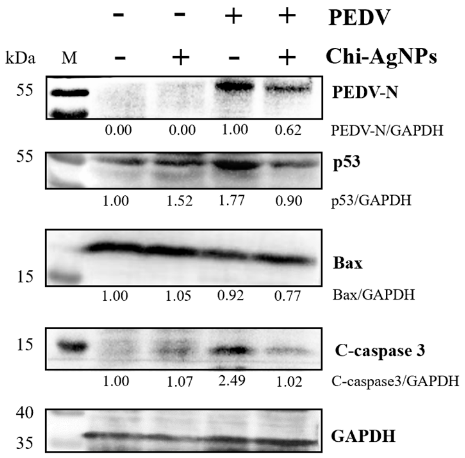

Fig. 9B Chi-AgNPs reduced PEDV causes cell apoptosis through activation of p53-mediated apoptotic pathway.
